# Supplementary material for: Multi-species single-cell transcriptomic analysis of ocular compartment regulons
Source: Nat Commun. 2021 Sep 28;12:5675. doi: 10.1038/s41467-021-25968-8 (PMC8478974; doi:10.1038/s41467-021-25968-8)
Supplement: Supplementary file 1 — Supplementary Information [file 41467_2021_25968_MOESM1_ESM.pdf]

Supplementary Information

Multi-species single-cell transcriptomic analysis of ocular compartment regulons

Pradeep Gautam, Kiyofumi Hamashima, Ying Chen, Yingying Zeng, Bar Makovoz, Bhav Harshad Parikh, Hsin Yee Lee, Katherine Anne Lau, Xinyi Su, Raymond CB Wong, Woon-Khiong Chan, Hu Li*, Timothy A Blenkinsop*, Yuin-Han Loh*

* Correspondence to: Li.Hu@mayo.edu, timothy.blenkinsop@mssm.edu, or yhloh@imcb.a-star.edu.sg

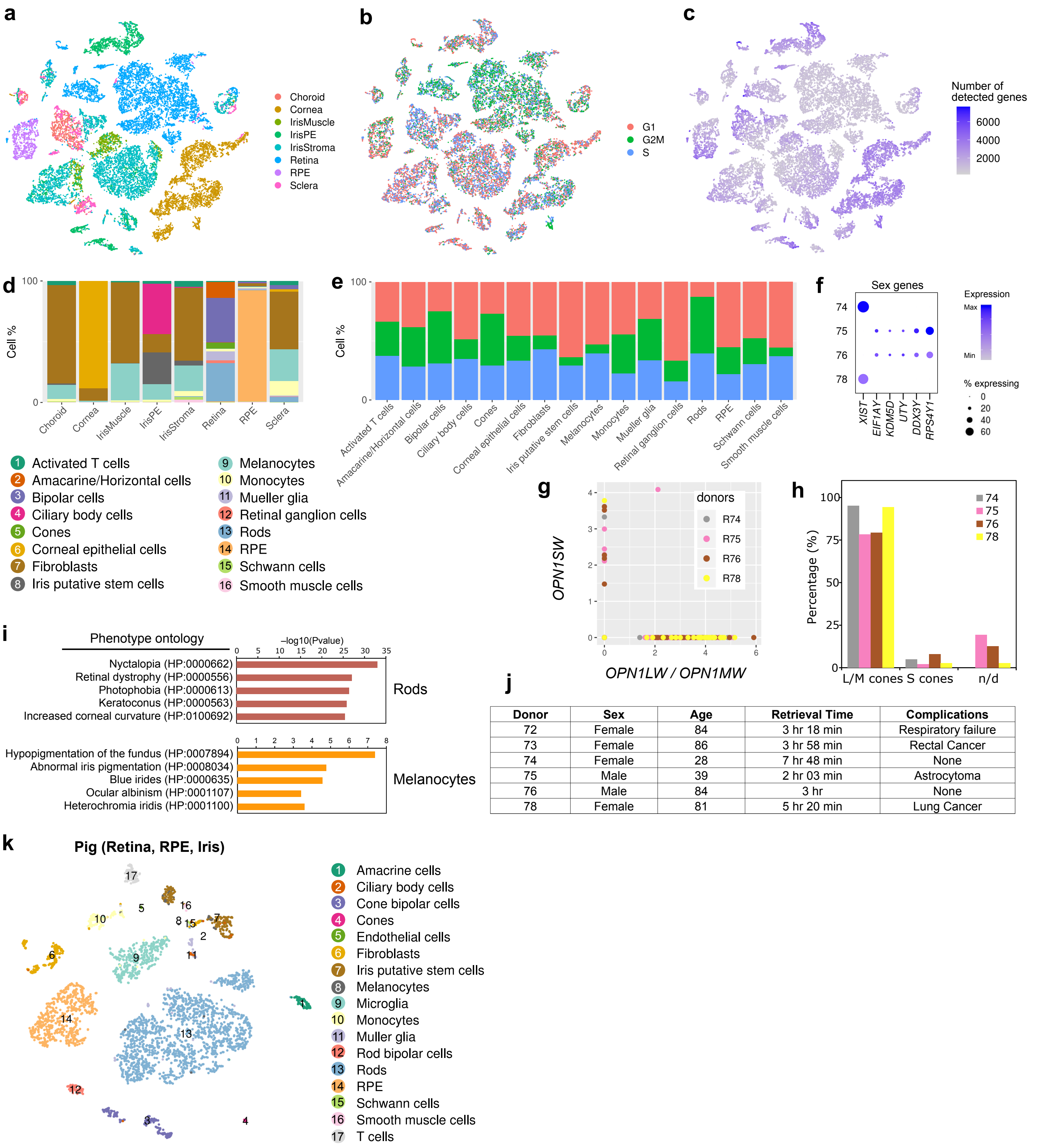

Donor	Sex	Age	Retrieval Time	Complications
72	Female	84	3 hr 18 min	Respiratory failure
73	Female	86	3 hr 58 min	Rectal Cancer
74	Female	28	7 hr 48 min	None
75	Male	39	2 hr 03 min	Astrocytoma
76	Male	84	3 hr	None
78	Female	81	5 hr 20 min	Lung Cancer

Figure S1

Supplementary Figure 1: Data quality post-processing and pig single-cell atlas

a-c. tSNE plot visualization of human eye cells coloured by tissues **(a)** that were dissected from the human eye, cell-cycle phases **(b)** and the number of detected genes **(c)** counted from individual single cells.

d. Proportion of cell types present in each tissue.

e. Proportion of cell cycle phases in each cell type.

f. Bubble plot showing expression of Y chromosome and maternal X chromosome-linked genes. Rows contain the donor names, while columns represent genes.

g. Scatter plot showing normalized expression values of *OPN1LW/OPN1MW* (x-axis) and *OPN1SW* (y-axis) for all cone cells identified in each donor.

h. Percentages of cone cells having either S or L/M wavelength cones.

i. Enriched phenotype ontologies in DEGs of Rod PR or Melanocytes. The phenotype ontology was obtained from modPheEA. Fisher's exact tests were applied to obtain a p-value for each phenotype based on the null hypothesis.

j. Information about donors whose eye cells were used for scRNA-seq experiments.

k. tSNE plot visualization of pig retina, RPE and iris cells.

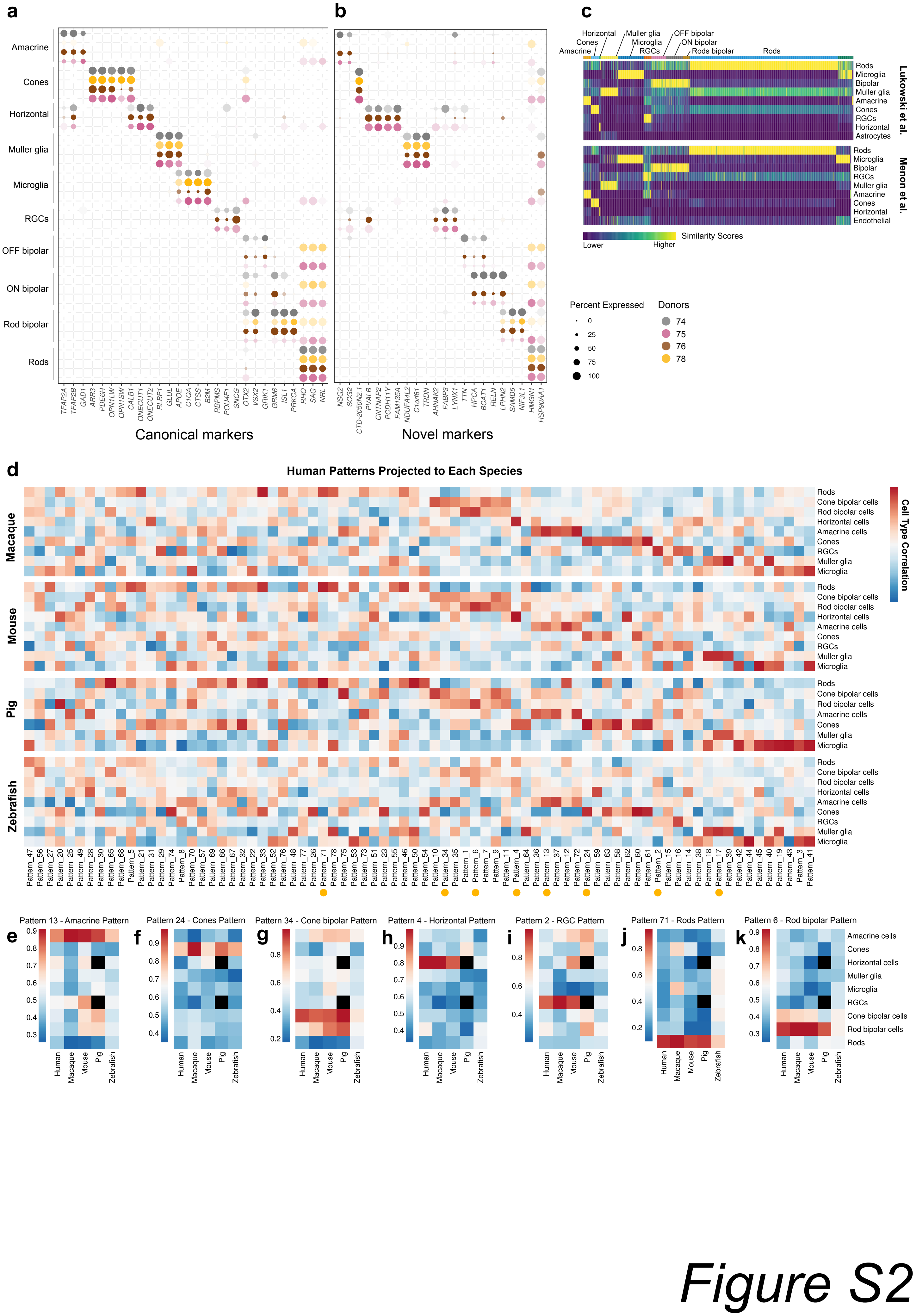
a
b
c
Canonical markers
Novel markers
Amacrine
Cones
Horizontal
Muller glia
Microglia
RGCs
OFF bipolar
ON bipolar
Rod bipolar
Rods
TFAP2A TFAP2B GAD1 ARR3 PDE6H OPN1LW OPN1SW CALB1 ONECUT1 ONECUT2 RLBP1 GLUL APOE C1QA CTSS B2M RBPMS POU4F1 SNCG OTX2 VSX2 GRIK1 GRM6 ISL1 PRKCA RHO SAG NRL
NSG2 SCG2 CTD-2050N2.1 PVALB CNTNAP2 PCDH11Y FAM135A NDUFA4L2 C1orf61 TRDN AHNAK2 FABP3 LYNX1 TTN HPCA BCAT1 RELN LPHN2 SAMD5 NIF3L1 HMGN1 HSP90AA1
Lukowski et al.
Menon et al.
Similarity Scores
Lower
Higher
Percent Expressed
0
25
50
75
100
Donors
74
75
76
78
d
Human Patterns Projected to Each Species
Macaque
Mouse
Pig
Zebrafish
Cell Type Correlation
Cone bipolar cells
Rod bipolar cells
Horizontal cells
Amacrine cells
Muller glia
e
f
g
h
i
j
k
Pattern 13 - Amacrine Pattern
Pattern 24 - Cones Pattern
Pattern 34 - Cone bipolar Pattern
Pattern 4 - Horizontal Pattern
Pattern 2 - RGC Pattern
Pattern 71 - Rods Pattern
Pattern 6 - Rod bipolar Pattern
Human
Macaque
Mouse
Pig
Zebrafish
Figure S2

Supplementary Figure 2: Characterization of cell types among retina across donor/species

a. Bubble plot showing expression of canonical markers specific for distinct cell-types in neural retinal layers of the eye. The size of each circle is proportional to the percentage of cells expressing the gene, and its intensity depicts the average transcript count within expressing cells.

b. Bubble plot showing expression of novel markers specific for distinct cell types in neural retinal layers of the eye. The size of each circle is proportional to the percentage of cells expressing the gene, and its intensity depicts the average transcript count within expressing cells.

c. Comparison of the expression of cone cell markers between our study and Lukowski et al. and Menon et. al.

d. The patterns of gene expression in human retinal cells projected into cross-species retinal cell types.

e-k. Specificity of Pattern 13 in Amacrine cells, Pattern 24 in Cone cells, Pattern 34 for Cone bipolar cells, Pattern 4 for Horizontal cells, Pattern 2 for RGC cells, Pattern 71 for Rod cells, and Pattern 6 for Rod bipolar cells across species.

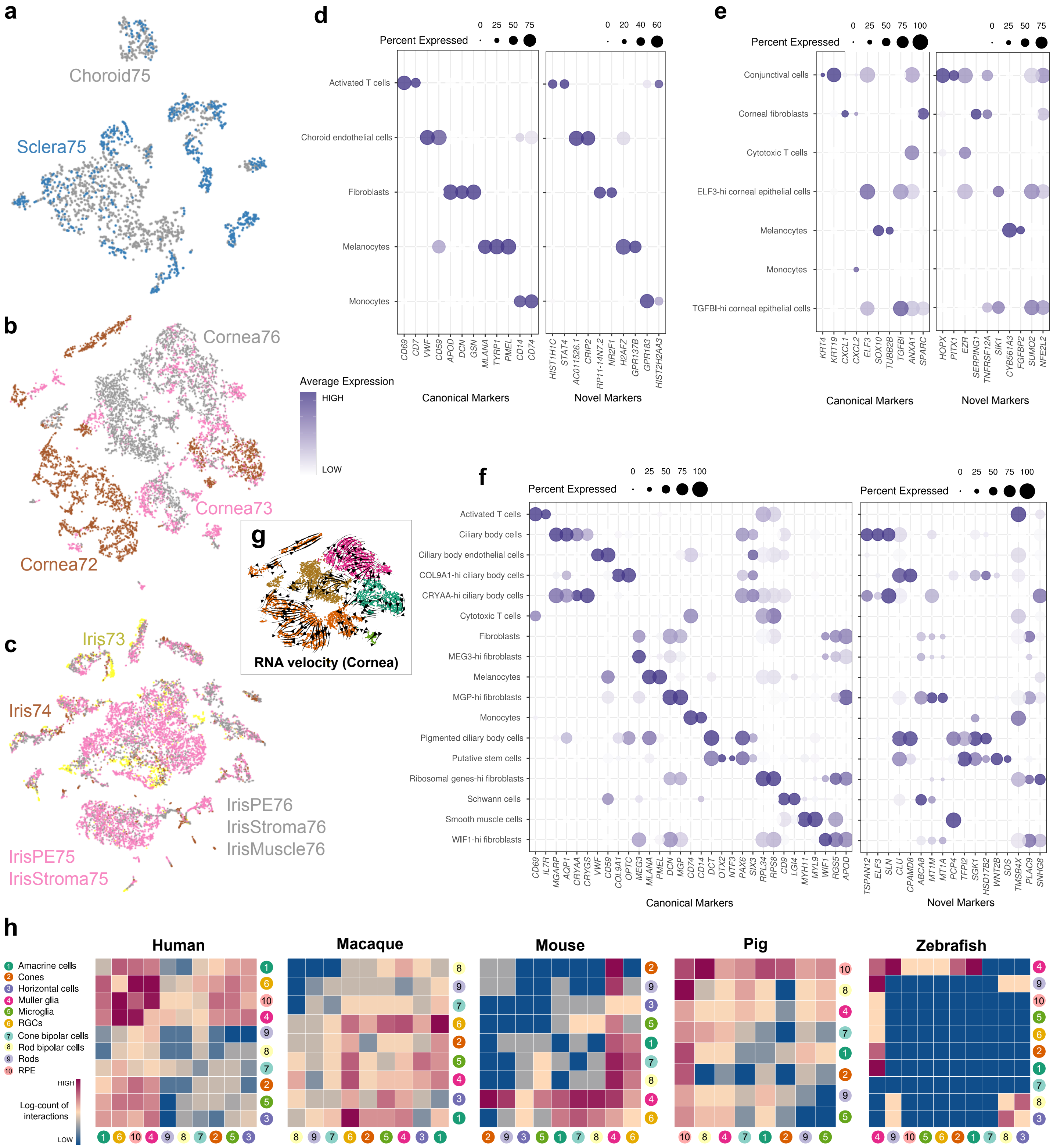
a
Choroid75
Sclera75
b
Cornea76
Cornea73
Cornea72
c
Iris73
Iris74
IrisPE76
IrisStroma76
IrisMuscle76
IrisPE75
IrisStroma75
d
Percent Expressed
Activated T cells
Choroid endothelial cells
Fibroblasts
Melanocytes
Monocytes
CD69 CD7 VWF CD59 APOD DCN GSN MLANA TYRP1 PMEL CD14 CD74
HIST1H1C STAT4 AC011526.1 CRIP2 RP11-14N7.2 NR2F1 H2AFZ GPR137B GPR183 HIST2H2AA3
Canonical Markers
Novel Markers
Average Expression
HIGH
LOW
e
Percent Expressed
Conjunctival cells
Corneal fibroblasts
Cytotoxic T cells
ELF3-hi corneal epithelial cells
Melanocytes
Monocytes
TGFBI-hi corneal epithelial cells
KRT4 KRT19 CXCL1 CXCL2 ELF3 SOX10 TUBB2B TGFBI ANXA1 SPARC
HOPX PITX1 EZR SERPING1 TNFRSF12A SIK1 CYB561A3 FGFBP2 SUMO2 NFE2L2
Canonical Markers
Novel Markers
f
Percent Expressed
Activated T cells
Ciliary body cells
Ciliary body endothelial cells
COL9A1-hi ciliary body cells
CRYAA-hi ciliary body cells
Cytotoxic T cells
Fibroblasts
MEG3-hi fibroblasts
Melanocytes
MGP-hi fibroblasts
Monocytes
Pigmented ciliary body cells
Putative stem cells
Ribosomal genes-hi fibroblasts
Schwann cells
Smooth muscle cells
WIF1-hi fibroblasts
CD69 IL7R MGARP AQP1 CRYAA CRYGS VWF CD59 COL9A1 OPTC MEG3 MLANA PMEL DCN MGP CD74 CD14 DCT OTX2 NTF3 PAX6 SIX3 RPL34 RPS8 CD9 LGI4 MYH11 MYL9 WIF1 RGS5 APOD
TSPAN12 ELF3 SLN CLU CPAMD8 ABCA8 MT1M MT1A PCP4 TFPI2 SGK1 HSD17B2 WNT2B SDS TMSB4X PLAC9 SNHG8
Canonical Markers
Novel Markers
g
RNA velocity (Cornea)
h
Human
Macaque
Mouse
Pig
Zebrafish
1 Amacrine cells
2 Cones
3 Horizontal cells
4 Muller glia
5 Microglia
6 RGCs
7 Cone bipolar cells
8 Rod bipolar cells
9 Rods
10 RPE
HIGH
Log-count of interactions
LOW

Figure S3

Supplementary Figure 3: Diversity of cell types in non-retinal cells of the eye

a,b,c. tSNE plot visualization of cell types from choroid/sclera layers **(a)**, cornea**(b)** and different regions of iris **(c)**.

d,e,f. Bubble plot showing expression of canonical and novel markers specific for distinct cell types in scleral/choroidal layers **(d)**, the cornea **(e)** and different regions of the iris **(f)**. The size of each circle is proportional to the percentage of cells expressing the gene, and its intensity depicts the average transcript count within expressing cells.

g. RNA velocity in corneal cells showing the process of inflammation and wound healing.

h. Muller glial cells have higher interaction with other retinal cell types across species.

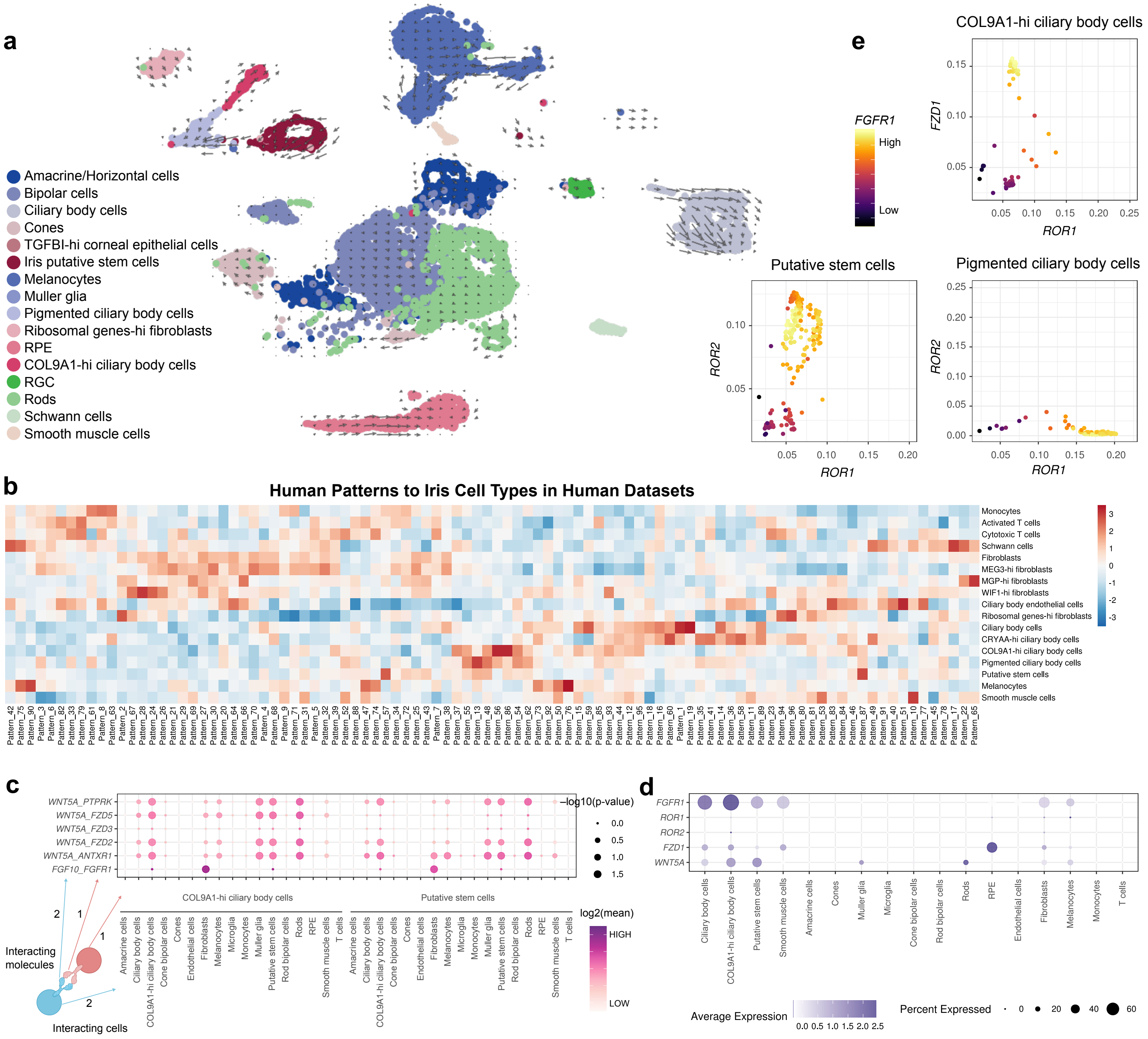
a
Amacrine/Horizontal cells
Bipolar cells
Ciliary body cells
Cones
TGFBI-hi corneal epithelial cells
Iris putative stem cells
Melanocytes
Muller glia
Pigmented ciliary body cells
Ribosomal genes-hi fibroblasts
RPE
COL9A1-hi ciliary body cells
RGC
Rods
Schwann cells
Smooth muscle cells
e
FGFR1
High
Low
COL9A1-hi ciliary body cells
FZD1
ROR1
Putative stem cells
ROR2
Pigmented ciliary body cells
b
Human Patterns to Iris Cell Types in Human Datasets
Monocytes
Activated T cells
Cytotoxic T cells
Schwann cells
Fibroblasts
MEG3-hi fibroblasts
MGP-hi fibroblasts
WIF1-hi fibroblasts
Ciliary body endothelial cells
Ribosomal genes-hi fibroblasts
Ciliary body cells
CRYAA-hi ciliary body cells
COL9A1-hi ciliary body cells
Pigmented ciliary body cells
Putative stem cells
Melanocytes
Smooth muscle cells
c
WNT5A_PTPRK
WNT5A_FZD5
WNT5A_FZD3
WNT5A_FZD2
WNT5A_ANTXR1
FGF10_FGFR1
-log10(p-value)
COL9A1-hi ciliary body cells
Putative stem cells
log2(mean)
HIGH
LOW
Interacting molecules
Interacting cells
Amacrine cells
Ciliary body cells
COL9A1-hi ciliary body cells
Cone bipolar cells
Cones
Endothelial cells
Fibroblasts
Melanocytes
Microglia
Monocytes
Muller glia
Putative stem cells
Rod bipolar cells
Rods
RPE
Smooth muscle cells
T cells
d
FGFR1
ROR1
ROR2
FZD1
WNT5A
Ciliary body cells
COL9A1-hi ciliary body cells
Putative stem cells
Smooth muscle cells
Amacrine cells
Cones
Muller glia
Microglia
Cone bipolar cells
Rod bipolar cells
Rods
RPE
Endothelial cells
Fibroblasts
Melanocytes
Monocytes
T cells
Average Expression
Percent Expressed

Figure S4

Supplementary Figure 4: Understanding putative stem cells of the eye

a. RNA velocity analysis shows that putative stem cells, pigmented ciliary body cells and

COL9A1 high ciliary body cells cluster together. The velocity arrows show putative stem cells could be cells of origin for the other two cell types.

b. Projection of patterns specific to human iris cell types.

c. Interaction map between several WNTs with different cells of the eye in the pig. The p-value for the likelihood of cell-type specificity of a given receptor-ligand complex is calculated based on the proportion of the means that are as high as or higher than the actual mean.

d. Expression of several FGFs with different cells of pig eye.

e. Co-expression analysis of *ROR1*, *ROR2*, and *FZD1* based on MAGIC imputation, showing these receptors do not express together in a single cell while all of them show co-occupancy with *FGFR1*.

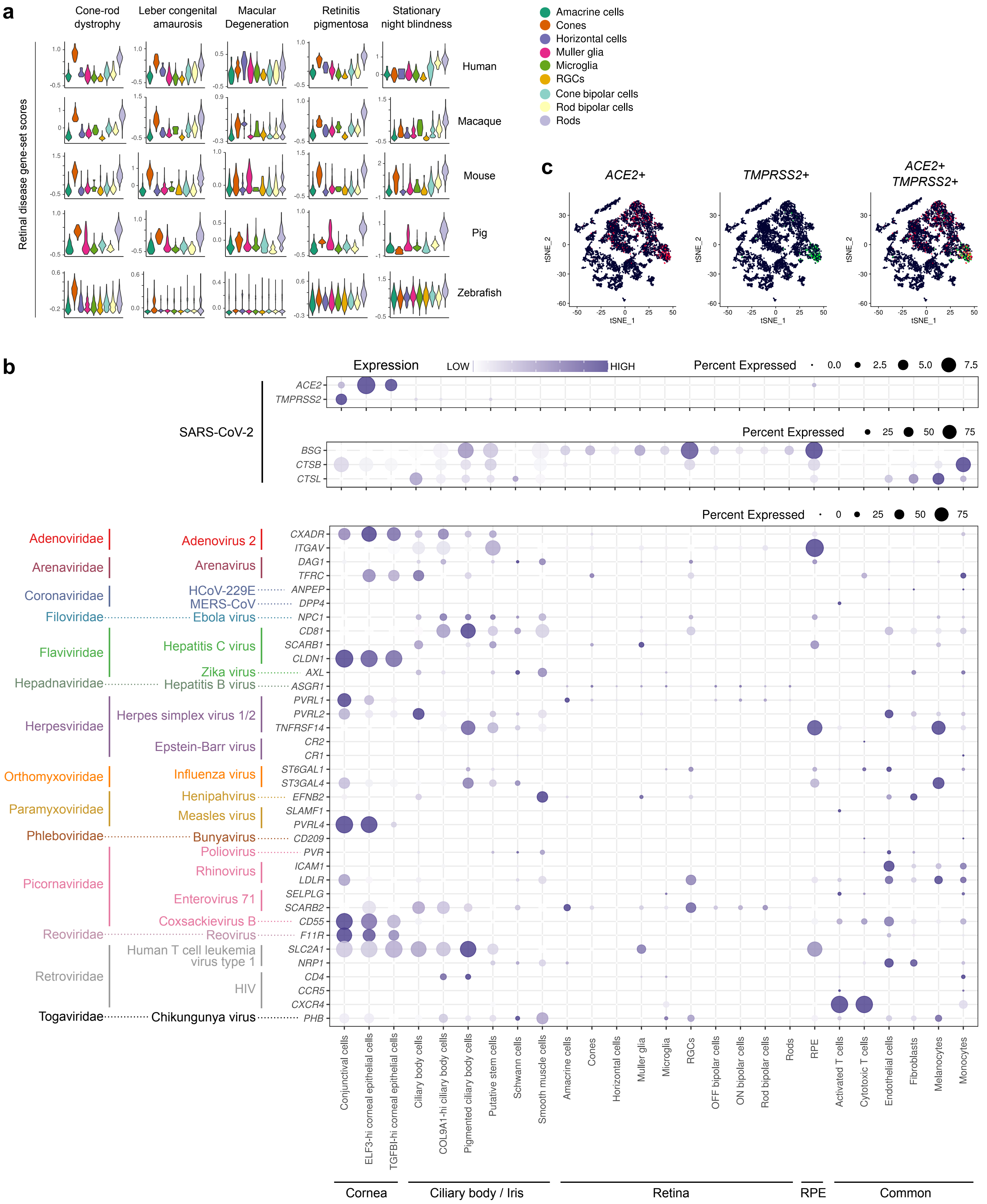
a
Cone-rod dystrophy
Leber congenital amaurosis
Macular Degeneration
Retinitis pigmentosa
Stationary night blindness
Retinal disease gene-set scores
Human
Macaque
Mouse
Pig
Zebrafish
Amacrine cells
Cones
Horizontal cells
Muller glia
Microglia
RGCs
Cone bipolar cells
Rod bipolar cells
Rods
c
ACE2+
TMPRSS2+
ACE2+ TMPRSS2+
tSNE_1
tSNE_2
b
Expression
LOW
HIGH
Percent Expressed
SARS-CoV-2
ACE2
TMPRSS2
BSG
CTSB
CTSL
Adenoviridae
Adenovirus 2
Arenaviridae
Arenavirus
Coronaviridae
HCoV-229E
MERS-CoV
Filoviridae
Ebola virus
Flaviviridae
Hepatitis C virus
Zika virus
Hepadnaviridae
Hepatitis B virus
Herpesviridae
Herpes simplex virus 1/2
Epstein-Barr virus
Orthomyxoviridae
Influenza virus
Paramyxoviridae
Henipahvirus
Measles virus
Phleboviridae
Bunyavirus
Picornaviridae
Poliovirus
Rhinovirus
Enterovirus 71
Coxsackievirus B
Reoviridae
Reovirus
Retroviridae
Human T cell leukemia virus type 1
HIV
Togaviridae
Chikungunya virus
CXADR
ITGAV
DAG1
TFRC
ANPEP
DPP4
NPC1
CD81
SCARB1
CLDN1
AXL
ASGR1
PVRL1
PVRL2
TNFRSF14
CR2
CR1
ST6GAL1
ST3GAL4
EFNB2
SLAMF1
PVRL4
CD209
PVR
ICAM1
LDLR
SELPLG
SCARB2
CD55
F11R
SLC2A1
NRP1
CD4
CCR5
CXCR4
PHB
Conjunctival cells
ELF3-hi corneal epithelial cells
TGFBI-hi corneal epithelial cells
Ciliary body cells
COL9A1-hi ciliary body cells
Pigmented ciliary body cells
Putative stem cells
Schwann cells
Smooth muscle cells
Amacrine cells
Cones
Horizontal cells
Muller glia
Microglia
RGCs
OFF bipolar cells
ON bipolar cells
Rod bipolar cells
Rods
RPE
Activated T cells
Cytotoxic T cells
Endothelial cells
Fibroblasts
Melanocytes
Monocytes
Cornea
Ciliary body / Iris
Retina
RPE
Common

Figure S5

Supplementary Figure 5: Creating a disease map across species and viral entry map in human eye cell types

a. Disease map of genes involved in different eye disorders checked across species.

b. Bubble plot of genes that act as receptors for virus entry into human cells across eye cell types. The size of each circle is proportional to the percentage of cells expressing the gene, and its intensity depicts the average transcript count within expressing cells.

c. tSNE plot of genes that act as receptors for SARS-CoV-2 across human eye atlas.

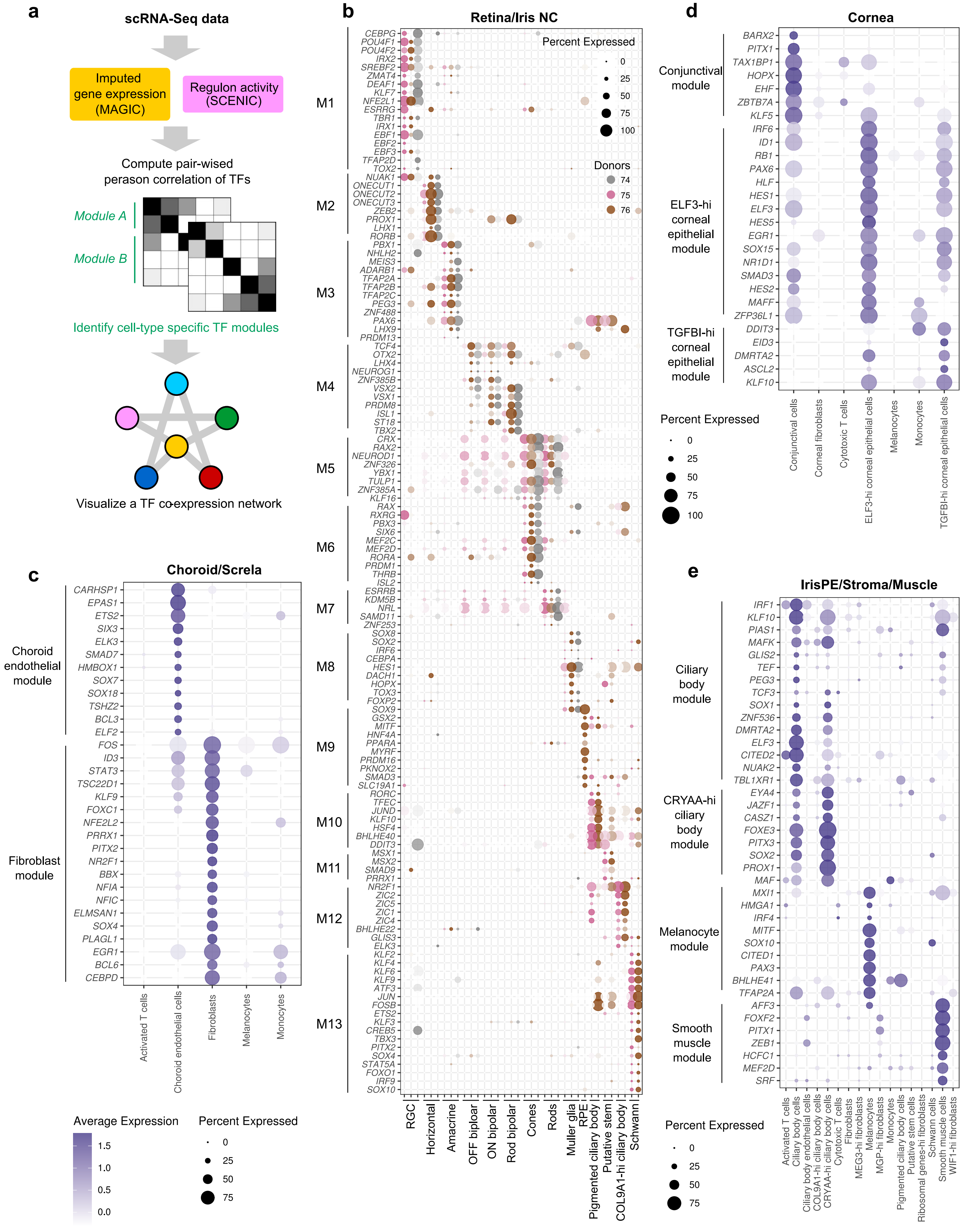
a
scRNA-Seq data
Imputed gene expression (MAGIC)
Regulon activity (SCENIC)
Compute pair-wised perason correlation of TFs
Module A
Module B
Identify cell-type specific TF modules
Visualize a TF co-expression network
b
Retina/Iris NC
Percent Expressed
0
25
50
75
100
Donors
74
75
76
M1
M2
M3
M4
M5
M6
M7
M8
M9
M10
M11
M12
M13
CEBPG
POU4F1
POU4F2
IRX2
SREBF2
ZMAT4
DEAF1
KLF7
NFE2L1
ESRRG
TBR1
IRX1
EBF1
EBF2
EBF3
TFAP2D
TOX2
NUAK1
ONECUT1
ONECUT2
ONECUT3
ZEB2
PROX1
LHX1
RORB
PBX1
NHLH2
MEIS3
ADARB1
TFAP2A
TFAP2B
TFAP2C
PEG3
ZNF488
PAX6
LHX9
PRDM13
TCF4
OTX2
LHX4
NEUROG1
ZNF385B
VSX2
VSX1
PRDM8
ISL1
ST18
TBX2
CRX
RAX2
NEUROD1
ZNF326
YBX1
TULP1
ZNF385A
KLF16
RAX
RXRG
PBX3
SIX6
MEF2C
MEF2D
RORA
PRDM1
THRB
ISL2
ESRRB
KDM5B
NRL
SAMD11
ZNF253
SOX8
SOX2
IRF6
CEBPA
HES1
DACH1
HOPX
TOX3
FOXP2
SOX9
GSX2
MITF
HNF4A
PPARA
MYRF
PRDM16
PKNOX2
SMAD3
SLC19A1
RORC
TFEC
JUND
KLF10
HSF4
BHLHE40
DDIT3
MSX1
MSX2
SMAD9
PRRX1
NR2F1
ZIC2
ZIC5
ZIC1
ZIC4
BHLHE22
GLIS3
ELK3
KLF2
KLF4
KLF6
KLF9
ATF3
JUN
FOSB
ETS2
KLF3
CREB5
TBX3
PITX2
SOX4
STAT5A
FOXO1
IRF9
SOX10
RGC
Horizontal
Amacrine
OFF biploar
ON bipolar
Rod bipolar
Cones
Rods
Muller glia
RPE
Pigmented ciliary body
Putative stem
COL9A1-hi ciliary body
Schwann
c
Choroid/Screla
Choroid endothelial module
Fibroblast module
CARHSP1
EPAS1
ETS2
SIX3
ELK3
SMAD7
HMBOX1
SOX7
SOX18
TSHZ2
BCL3
ELF2
FOS
ID3
STAT3
TSC22D1
KLF9
FOXC1
NFE2L2
PRRX1
PITX2
NR2F1
BBX
NFIA
NFIC
ELMSAN1
SOX4
PLAGL1
EGR1
BCL6
CEBPD
Activated T cells
Choroid endothelial cells
Fibroblasts
Melanocytes
Monocytes
Average Expression
1.5
1.0
0.5
0.0
Percent Expressed
0
25
50
75
d
Cornea
Conjunctival module
ELF3-hi corneal epithelial module
TGFBI-hi corneal epithelial module
BARX2
PITX1
TAX1BP1
HOPX
EHF
ZBTB7A
KLF5
IRF6
ID1
RB1
PAX6
HLF
HES1
ELF3
HES5
EGR1
SOX15
NR1D1
SMAD3
HES2
MAFF
ZFP36L1
DDIT3
EID3
DMRTA2
ASCL2
KLF10
Conjunctival cells
Corneal fibroblasts
Cytotoxic T cells
ELF3-hi corneal epithelial cells
Melanocytes
Monocytes
TGFBI-hi corneal epithelial cells
Percent Expressed
0
25
50
75
100
e
IrisPE/Stroma/Muscle
Ciliary body module
CRYAA-hi ciliary body module
Melanocyte module
Smooth muscle module
IRF1
KLF10
PIAS1
MAFK
GLIS2
TEF
PEG3
TCF3
SOX1
ZNF536
DMRTA2
ELF3
CITED2
NUAK2
TBL1XR1
EYA4
JAZF1
CASZ1
FOXE3
PITX3
SOX2
PROX1
MAF
MXI1
HMGA1
IRF4
MITF
SOX10
CITED1
PAX3
BHLHE41
TFAP2A
AFF3
FOXF2
PITX1
ZEB1
HCFC1
MEF2D
SRF
Activated T cells
Ciliary body cells
Ciliary body endothelial cells
COL9A1-hi ciliary body cells
CRYAA-hi ciliary body cells
Cytotoxic T cells
Fibroblasts
MEG3-hi fibroblasts
Melanocytes
MGP-hi fibroblasts
Monocytes
Pigmented ciliary body cells
Putative stem cells
Ribosomal genes-hi fibroblasts
Schwann cells
Smooth muscle cells
WIF1-hi fibroblasts
Percent Expressed
0
25
50
75

Figure S6

Supplementary Figure 6: Characterization of transcription factor modules of all cell types of eye

a. Schematic describing the method to generate cell type-specific TF module.

b-e. Bubble plot showing expression of TFs grouped by the identified modules in neuronal/glial cells (b), sclera/choroid cells (c), corneal cells (d) and iris cells (e) in the eye. The size of each circle is proportional to the percentage of cells expressing the gene, and its intensity depicts the average transcript count within expressing cells.

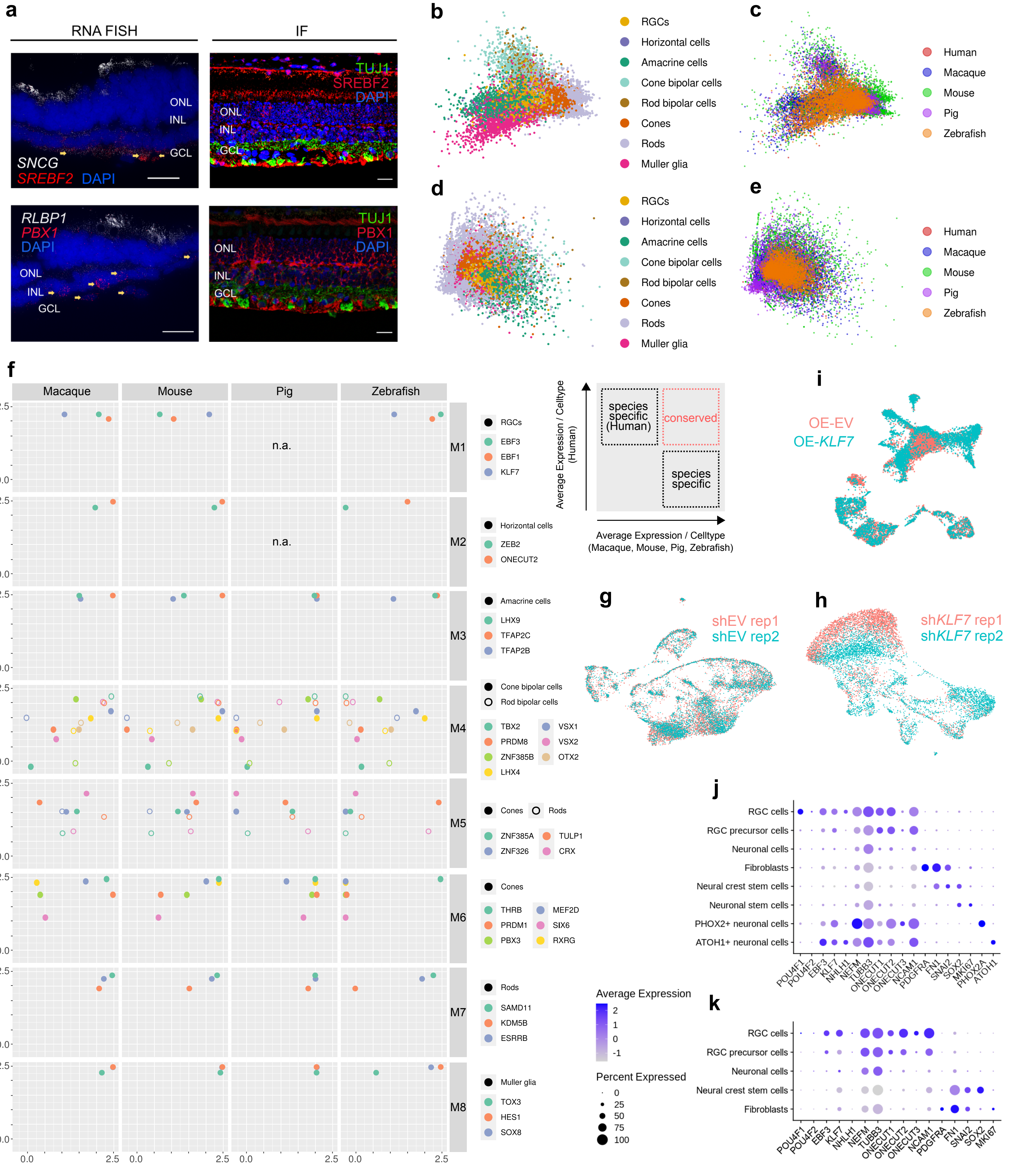

a
RNA FISH
IF
ONL
INL
GCL
SNCG
SREBF2 DAPI
TUJ1
SREBF2
DAPI
RLBP1
PBX1
DAPI
TUJ1
PBX1
DAPI
b
RGCs
Horizontal cells
Amacrine cells
Cone bipolar cells
Rod bipolar cells
Cones
Rods
Muller glia
c
Human
Macaque
Mouse
Pig
Zebrafish
d
e
f
Macaque
Mouse
Pig
Zebrafish
n.a.
M1
M2
M3
M4
M5
M6
M7
M8
2.5
0.0
RGCs
EBF3
EBF1
KLF7
Horizontal cells
ZEB2
ONECUT2
Amacrine cells
LHX9
TFAP2C
TFAP2B
Cone bipolar cells
Rod bipolar cells
TBX2
PRDM8
ZNF385B
LHX4
VSX1
VSX2
OTX2
Cones
Rods
ZNF385A
ZNF326
TULP1
CRX
THRB
PRDM1
PBX3
MEF2D
SIX6
RXRG
SAMD11
KDM5B
ESRRB
Muller glia
TOX3
HES1
SOX8
Average Expression / Celltype (Human)
species specific (Human)
conserved
species specific
Average Expression / Celltype (Macaque, Mouse, Pig, Zebrafish)
i
OE-EV
OE-KLF7
g
shEV rep1
shEV rep2
h
shKLF7 rep1
shKLF7 rep2
j
RGC cells
RGC precursor cells
Neuronal cells
Fibroblasts
Neural crest stem cells
Neuronal stem cells
PHOX2+ neuronal cells
ATOH1+ neuronal cells
POU4F1
POU4F2
EBF3
KLF7
NHLH1
NEFM
TUBB3
ONECUT1
ONECUT2
ONECUT3
NCAM1
PDGFRA
FN1
SNAI2
SOX2
MKI67
PHOX2A
ATOH1
Average Expression
2
1
0
-1
Percent Expressed
0
25
50
75
100
k

Figure S7

Supplementary Figure 7: Understanding role of KLF7 in RGC maturation

a. RNA FISH of PBX1 and SREBF2 showing the localization in INL layer and GCL layer respectively. n=2 technical replicates. Immunostaining of PBX1 and SREBF2 with TUJ1 in non-human primate samples. n=2 technical replicates. Scale bar = 20 um.

b,c. PCA-dimension reduction of expression profiles of 31 genes in M1-M9 modules, showing sufficient segregation between different cell types of the retina across species.

d,e. PCA-dimension reduction of expression profiles of randomly selected 31 TFs as a negative control.

f. Checking conservation of TFs by plotting pairwise correlation across species.

g. UMAP plot visualization of two replicates for cells obtained from differentiating RGC cells transfected with empty vector as control.

h. UMAP plot visualization of two replicates for cells obtained from differentiating RGC cells transfected with s*hKLF7*.

i. UMAP plot visualization of two conditions for cells obtained from differentiating RGC cells in overexpression experiments.

j,k. Bubble plot of genes used for annotation in KD and OE libraries. The size of each circle is proportional to the percentage of cells expressing the gene, and its intensity depicts the average transcript count within expressing cells.

Supplementary Methods

Pre-processing of single-cell RNA-seq data

Alignment to the reference genome, quantification and initial quality control (QC) were performed using the 10X Genomics' cellranger' pipeline (version >2.1.1)[1] and the R package 'Seurat' (version >3.0.1)[2] (https://github.com/satijalab/seurat). We used 'cellranger mkfastq' to generate fastq files from raw Illumina BCL files and 'cellranger count' to generate read count matrices from the fastq files. The reference genome index of hg38 and suscr11 was used for humans and pigs respectively, with the parameters'--force-cells=6000' for donor 75 retina and '--expect-cells=6000' for the others. Count data were imported into Seurat objects, and the QC was performed to remove outlier cells and genes in five steps. First, cells expressing < 15% of mitochondrial genes for all primary tissues datasets and < 10% for OE-EV; <20% for OE-KLF7; <10% for shEV-I, shEV-II, shKLF7_I, and shKLF7_II for *in vitro* RGC differentiation datasets were retained. Second, cells with the following numbers of detected genes were retained: 200-8,000 for human donor 74 retina; >200 for human donor 75 retina; 200-4,000 for human donor 76 retina; 200-7,000 for human donor 78 retina; 200-5,500 for human donor 76 RPE; 200-4,000 for human donor 78 RPE; 200-7500 for human donor 73 iris; 200-7500 for human donor 74 iris; 200-5,000 for human donor 75 iris PE; 200-7,500 for human donor 76 iris PE; 200-4,000 for human donor 75 iris stroma; 200-5,000 for human donor 76 iris stroma; 200-3,700 for human donor 76 iris muscle; 200-5,000 for human donor 75 sclera; 200-4,500 for human donor 75 choroid; >200 for human donor 72 cornea; 200-8,500 for human donor 73 cornea; 200-6,000 for human donor 76 cornea; <2,500 for pig retina; <3,500 for pig RPE; <4,000 for pig iris; 500-6,000 for OE-EV; 500-7,500 for OE-KLF7; 500-6,000 for shEV_I, shEV_II, shKLF7_I and shKLF7_II for *in vitro* RGC differentiation. Third, cells with less than the following numbers of total UMIs were retained: 40K for human donor 74 retina; 10K for human donor 76 retina; 35K for human donor 78 retina; 20K for human donor 76 RPE; 12K for human donor 78 RPE; 30K for human donor 73 iris; 50K for human donor 74 iris; 20K for human donor 75 iris PE; 30K for human donor 76 iris PE; 14K for human donor 75 iris stroma; 15K for human donor 76 iris stroma; 12K for human donor 76 iris muscle; 20K for human donor 75 sclera; 20K for human donor 75 choroid; 100K for human donor 72 cornea; 60K for human donor 73 cornea; 30K for human donor 76 cornea; 5K for pig retina; 10K for pig RPE; 10K for pig iris; 35K for OE-EV; 50K for OE-KLF7; 50K for shEV_I, shEV_II, shKLF7_I and shKLF7_II for *in vitro* RGC differentiation. Fourth, we identified doublets using by the python package 'Scrublet'[3] (https://github.com/AllonKleinLab/scrublet) with the default

parameters and cells with less than following doublet scores were retained: 0.16 for human donor 74 retina; 0.20 for human donor 75 retina; 0.20 for human donor 76 retina; 0.20 for human donor 78 retina; 0.30 for human donor 76 RPE; 0.40 for human donor 78 RPE; 0.20 for human donor 73 iris; 0.20 for human donor 74 iris; 0.20 for human donor 75 iris PE; 0.20 for human donor 76 iris PE; 0.20 for human donor 75 iris stroma; 0.20 for human donor 76 iris stroma; 0.20 for human donor 76 iris muscle; 0.14 for human donor 75 sclera; 0.19 for human donor 75 choroid; 0.20 for human donor 72 cornea; 0.23 for human donor 73 cornea; 0.23 for human donor 76 cornea; 0.22 for pig retina and 0.18 for pig iris. Fifth, genes were retained in the data if they were expressed in ≥ 3 cells. The Seurat' NormalizeData' function was used to perform an additional cell-cell normalisation, and the Seurat 'merge' function was used to combine the multiple libraries for each analysis. For public single-cell RNA-seq datasets, read count matrices of one human retina studies[4] were similarly generated from the downloaded fastq files. Read count matrices of the macaque retina study[5] were generated from the downloaded bam files with the macFas5 genome's index prepared using the author's compiled GTF file. Processed read count matrices of the mouse retina study[6], zebrafish retina study[7] and another human retina study[8] were downloaded from the Gene Expression Omnibus (GEO). The resulting read count matrices were further processed according to each author's procedures with slight modifications.

Dimensionality reduction, clustering and visualisation

The downstream analyses were also performed mainly by the Seurat. The inherent variation caused by mitochondrial gene expression was regressed by the 'ScaleData' or 'SCTransform' function. To identify cell clusters, principle component analysis (PCA) was first performed on the list of genes with higher variation than expected (highly variable genes). The highly variable genes were identified with the 'FindVariableFeatures' function (selection.method = "vst") and selected with the following thresholds: >0.01 vst.mean, >1 vst.variance.standardized. The numbers of significant PCs used in each PCA were determined by the Jackstraw method and are as follows: 50 PCs for human mixed eye tissues; 40 PCs for human retina; 30 PCs for human iris; 20 PCs for human sclera/choroid; 30 PCs for human cornea; 30 PCs for pig mixed eye tissues; 10 PCs for *KLF7*-KD RGC; 10 PCs for *KLF7*-OE RGC. The Louvain algorithm-based clustering was then performed to generate cell clusters, using the 'FindNeighbors' and 'FindClusters' function with optimised 'resolution' values for each analysis. Lastly, the clustering results were visualised using t-distributed stochastic neighbour embedding (t-SNE) and Uniform Manifold Approximation and Projection (UMAP)

with optimised ‘n.neighbors’ and ‘spread’ parameters (in the ‘RunUMAP’ function) for each analysis. For the analysis of mixed donor datasets, except all primary tissues atlas (Fig 1), the batch differences were corrected before clustering using the Seurat ‘IntegrateData’ function with the same PC numbers determined by the Jackstraw method.

Prediction of the cell cycle phase

The Seurat ‘CellCycleScoring’ function was used to predict the cell cycle phase of individual cells. Briefly, S and G2/M scores were first assigned to each cell based on its expression of S and G2/M phase markers, previously defined[9]. The ‘CellCycleScoring’ function then predicts the classification of each cell in either G2/M, S or G1 phase. Finally, cells expressing neither of the G2/M or S phase markers were classified as being in the G1 phase.

Major cell class annotation

Major cell classes were manually annotated based on the cluster-specific markers. For the identification of cluster biomarkers, we used the Seurat ‘FindAllMarkers’ function (only.pos = TRUE, min.pct = 0.25, logfc.threshold = 0.25), which detects differentially expressed genes (DEG) for each cluster. Marker genes that were mainly used in our studies are listed in S Table 1. Note that the tissue name of the potentially contaminated cells was re-labelled as follows: ‘iris’ to ‘RPE’ for cells expressing RPE65 (normalised count >0.5); ‘RPE’ to ‘retina’ for cells with low RPE65 expression (normalised count <0.5) or high RHO expression (normalised count >3). The expression profile of the detected DEGs was plotted on the heatmap with the ‘DoHeatmap’ function. Some expressions were also visualised with the balloon plot (‘DotPlot’ function in Seurat) and plotted on the t-SNE/UMAP space (‘FeaturePlot’ function in Seurat). Gene set enrichment analysis (GSEA) of the DEGs for each cell type was carried out using Metascape[10] (http://metascape.org). Phenotype gene ontology (GO) was analysed using modPhEA[11] (http://evol.nhri.org.tw/phenome2/). Library similarities between human retinal single-cell studies were analysed using SingleR[12] (https://github.com/dviraran/SingleR).

Preparation of dataset and ortholog table used in cross-species comparison

Multiple single-cell datasets from human, macaque, mouse, pig and zebrafish were used in cross-species comparisons. Around One thousand three hundred human retina cells in the donor 74 samples, 4,396 macaque periphery retina cells in the M6 sample, 4,835 mouse retina cells

in the r3 sample, 2,550 pig retina cells, 4,552 zebrafish retina cells in the Adult R5 sample, 6,355 zebrafish Muller glial cells in the NMDA treatment samples, 51 human RPE cells in the donor 74 sample, 617 pig RPE cells, 67 zebrafish RPE cells in the Adult R5 sample, 9,047 human iris cells from the donor 75/76 and 734 pig iris cells were selected for this analysis. We targeted 8,831 common orthologs across five species selected using an Ensembl multiple species comparison tool (http://www.ensembl.org/biomart/martview/42ddd77f8b0f4aae7d9eefe32cc 4518c/). Each species was compared to humans, and a high-quality ortholog genes list was extracted (confidence score = 1). For paralogs, genes with the highest expression in ocular cells were selected. Each UMI count matrix was normalised using Seurat as described above and used for each downstream analysis.

scCoGAPS gene pattern analysis

To infer cell type-specific gene expression programs in the ocular tissues, 'scCoGAPS'[13] (version 3.6.0) (https://bioconductor.org/packages/release/bioc/vignettes/CoGAPS/inst/doc/CoGAPS.html) was used, which allows unsupervised identification of patterns of gene set usage that represent common features across cells. Human retinal cells, human iris cells or zebrafish retina cells were used as an input dataset that includes only orthologs selected as described above for human gene pattern estimation or all expressed genes for zebrafish gene pattern estimation. Mitochondrial and ribosomal protein-coding genes were excluded to reduce unexpected gene patterns that highlight sample batch effects. The parameters were default single-cell parameters, except nPatterns=100, nIterations=500, sparseOptimization = True, seed =830. The identified human retina, human iris and zebrafish retina patterns were projected into the expression matrix of other species using 'ProjectR' (version 1.5.0) to understand each pattern conservation. The pattern weights were tested for predictive power for each cell type annotation using ProjectR 'aucMat' function and visualised with blue-red scale heatmaps.

Cell type-cell type interaction analysis

To analyse crosstalks among all ocular cell-types in our atlas (related to Fig 3 and 4), 'CellPhoneDB'[14] (version 2.1.5) (https://github.com/Teichlab/cellphonedb) was used, which includes the information of interactions between ligands and receptors. The following parameters were used for analysis 'cellphonedb method statistical_analysis --threads=20 --counts-data hgnc_symbol'. To demonstrate an overview of potential cell-cell communications

(Fig 3d-f), highly variable interacting pairs (>4.5 coefficient of variation) were first selected from the list of interacting pairs which were identified in the down-sampled dataset (200 cells/cell type) and then visualised with the circos plot using the R package ‘circlize’. The interaction modules with <0.05 p-value, <0.05 p-value or <0.05 p-value & >0 mean in the Wnt, FGF and MDK signalling, respectively, were visualized with R package ggalluvial. Potential cell-cell interacting pairs in other species (macaque, mouse, pig and zebrafish) were similarly identified using the human cellphonedb database after converting each gene name into the corresponding human ortholog name as described above. Heatmaps were generated using the ‘cellphonedb plot heatmap_plot’ function.

Stem cell potency analysis

The R package ‘LandSCENT’[15] (https://github.com/ChenWeiyan/LandSCENT) was used to estimate the stem cell potency of every single cell in iris tissues. Non-corrected count matrix (assay ‘RNA’ in Seurat object) of only cells in donor 75 were selected as input data. The computation of SR values (stem cell potencies) was carried out by the ‘CompSRana’ function with default parameters and ‘net17Jan16.m’ as a protein-protein interaction database.

Estimating RNA velocity of cells

The python package ‘velocyto’[16] (http://velocyto.org/) was used for RNA-velocity analysis, which could estimate the RNA velocities of single cells through quantifying the differences between the unspliced and spliced mRNAs. UMAP embeddings produced by Seurat was used for the visualisation of RNA velocity.

Disease map generation

The list of eye disease genes was obtained from the NIH Genetics Home Reference (https://ghr.nlm.nih.gov). The list was not revised based on our own observations to ensure an unbiased representation. Only genes that are not expressed in our dataset were excluded from the disease map. Each disease gene-set score on the single-cell level was computed with Seurat ‘AddModuleScore’ function and plotted on the tSNE plot. Orthologs of human retinal disease genes in macaque, mouse, pig and zebrafish were collected from the Ensembl database as described above, and their module scores were analysed similarly. A list of viral receptors (used in SFig 5b) was drawn from the published papers[17,18].

Transcription factor module identification

Transcription factor modules of each cell type in the retina, iris, cornea and choroid/sclera tissues were identified based on the profile of gene expressions and the activity of the gene regulatory networks (regulons) (SFig 6a). First, missing values in the UMI count matrix were imputed by MAGIC[19] (https://github.com/KrishnaswamyLab/MAGIC), which denoise high-dimensional data, restoring the structure of large sparse count matrix of single-cell RNA-seq. After the running 'magic' function in R package 'Rmagic' against all genes with default parameters, the imputed count matrix was extracted for downstream analysis. Concurrently, regulon activities of each single-cell were computed by SCENIC[20] (https://github.com/aertslab/SCENIC), which reconstructs a single-cell regulatory network based on the co-expression patterning between TFs and potential target genes and the enrichment of the regulator's binding motif. SCENIC was carried out with default parameters and public cisTarget databases[21] (https://resources.aertslab.org/cistarget/). We confirmed the t-SNE analysis of the regulon activity matrix was consistent with the t-SNE analysis based on the UMI count matrix. The binarising process for the estimated regulon activities was omitted in this study. After filtering out regulons active in less than 7% of cells in the dataset, the regulon activity matrix was extracted for downstream analysis. Second, the pair-wised Pearson correlation coefficient among all TFs in each matrix was computed with R package 'cor' and visualised with 'corrplot'. TF modules were extracted from these two matrices by hierarchical clustering (hclust.method = 'word.D2') and finalised after some manual curations. Third, the co-expression network of the identified TF modules in the human retina and iris neuronal cells was visualised using Cytoscape[22] (version 3.7.1) with an edge-weighted spring embedded layout. The nodes represent cell types, and edges represent significant co-expressions.

Each TF module score on the single-cell level was computed with Seurat 'AddModuleScore' function and plotted on the tSNE plot. GSEA for each TF module was carried out using Metascape. In addition to TFs in each TF module, potential target genes of each TF, predicted by SCENIC, were included in the gene set. PCA was performed on the list of 31 TFs, which are typical among humans, macaque, mouse, pig and zebrafish. As a negative control, PCA was also performed on the list of randomly selected 31 TFs in triplicate to confirm the reproducibility of the analysis result.

Isolation of cell types from the human eye

Isolation of Cornea.

The anterior segment was separated from the rest of the eye by performing a circumferential incision 6mm posterior from the ora serrata. The lens, ciliary body and iris were then removed by manual manipulation using forceps. Conjunctiva was carefully dissected off of sclera. Cornea cells were isolated from the central part of the cornea leaving approximately 1mm distance from the edge of the transparent section of the anterior segment of the eye. This corneal tissue was then further cut into 1mm2 pieces and digested with collagenase 1mg/ml. The full-thickness was used, including corneal epithelium, corneal stroma and corneal endothelium. The limbal region was dissected from the remaining tissue after the cornea was removed. The limbus sample included the total thickness of 1mm of transparent cornea out of 1mm of opaque tissue.

Isolation of Sclera.

The sclera sample included the entire thickness of greater than 2mm away from the beginning of the opaque region of the anterior segment to 6mm of the opaque area.

Isolation of Ciliary body and Iris.

The ciliary body and iris were pulled away from the cornea/limbus/sclera tissue using #5 Dumont forceps. The iris was then gently pulled away from the ciliary body.

These separated tissues of the sclera, ciliary body and iris cells were then further cut into 1mm2 pieces and digested with collagenase 1mg/ml with 3ug/ml DNAse Solution (STEMCELL Technologies) in Earle’s balanced salt solution for 3 hours. Following digestion, tissue pieces were triturated for 2 minutes using a 10ml pipette. Finally, the tissue pieces are allowed to settle at the bottom of the conical tube, and the supernatant is collected, centrifuged and resuspended and frozen using CS2 medium (Cryostore).

Isolation of Retina.

Taking the posterior segment, the vitreous and retina was encouraged using angled forceps to separate from the back of the eyecup to the point where the retina was only still attached to the eye at the location of the eye the optic disk. Using micro scissors, the retina was cut from the optic disk. The vitreous was then separated from the retina using forceps. The whole retina was cut into approximately 1mm2 pieces and digested with hyaluronidase (6mg/ml) in Earle’s balanced salt solution for 3 hours. The tissue pieces are allowed to settle at the bottom of the conical tube, and the supernatant is collected, centrifuged, resuspended and frozen using CS2 medium (Cryostore).

Isolation of Retinal pigmented epithelium and choroid.

The posterior eyecup was placed in a cup with the optic nerve facing down. The eyecup is filled with 0.25% trypsin with 3ug/ml DNAse Solution and incubated at 37°C for 50 minutes. RPE was then brushed off the Bruch's Membrane, collected and frozen using CS2 medium. After the RPE are removed, choriocapillaris/Bruch's membrane is cut into 1mm2 pieces of tissue and placed into 2% collagenase 1mg/ml with 3ug/ml DNAse Solution. Afterwards, tissue pieces are triturated for 2 minutes using a 10ml pipette. The tissue pieces are allowed to settle at the bottom of the conical tube, and the supernatant is collected, centrifuged, resuspended, and frozen using a CS2 medium (Cryostore).

Isolation of RPE, Iris and Retina from Pig eye.

Pig eye samples were collected from Agri-Food and Veterinary Authority of Singapore approved abattoirs. The conditions of the abattoir was followed according to WHOLE MEAT AND FISH ACT enforced by Singapore Food Agency. Pig RPE, Iris and Retina was dissected with the same protocol described above for dissection in human eyes.

H9 cell culture and differentiation into retinal ganglion cells

Human ESCs (H9 [WA09, P35–50], WiCell, Madison, WI, USA) were cultured and maintained in MTers1 media (Stemcell Technologies) in Matrigel (Corning life sciences) coated plates. The protocol for RGC differentiation was adapted from Lee et al.[23]. Few changes were made to the protocol are described below. Embryoid bodies were made by dissociating H9 cells, which were 80 per cent confluent with Accutase (Innovative Cell Technologies) and resuspending them with Mtesr1 with 10 uM Y-27632 (Sigma Aldrich) in 96 well ultra-low attachment plates (Corning Costar). For differentiation toward RGCs, cells were seeded onto Matrigel-coated dishes instead of Poly-D-lysine and laminin-coated plates in the original protocol and cultured in N2B27 medium supplemented with 4 μM with N-(N-[3,5-difluorophenacetyl]-L-alanyl)-S-phenylglycine t-butyl ester (DAPT; Sigma-Aldrich) for ten days.

Transient transfection of RGC cells during differentiation

For transient transfection of RGC cells, sh*KLF7*I and sh*KLF7*II shRNAs were cloned in pSUPER puromycin plasmids. One microgram of plasmids was added to 180 ul of OptiMEM media (Thermofisher Technologies). It was mixed thoroughly by pipetting and incubated for 5

minutes at room temperature. 4 ul of Fugene HD (Promega) was added to 1 ug of plasmid and mixed and incubated at room temperature for 10 minutes. The mixture was then added to differentiating Retinal ganglion cell culture that was four days post addition of DAPT. The cells were treated with 0.15 uM of puromycin after 24 hours of treatment. The cells were harvested after 48 hours of transfection for single-cell RNAseq analysis. For overexpression studies, *KLF7* ORF was cloned into pCAG-puro plasmids. The cells were transfected into RGC cells before DAPT treatment and allowed to mature into RGC cells for six days and then harvested for single-cell RNAseq. The target sequences for sh*KLF7*I is GCTAGTTATAGTATATTCCA and sh*KLF7*II is GCCTTGAATTGGAACGCTA.

Immunofluorescence in Non- Human Primate Slides

Paraffin-embedded slides of non-human primate samples were used for immunofluorescence studies. They were deparaffinised by treatment with xylene and ethanol. After repeated washing of slides in decreasing ethanol concentration, the slides were subjected to antigen retrieval in a citrate buffer at pH 7.0. Next, the slides were blocked using a 1 % BSA PBST (PBS buffer +0.1% Triton X) blocking buffer. After that, they were incubated with primary antibodies overnight at 4°C. Anti-TUJ1 Ab18207(Abcam), Anti-SREBP2 AV09037(Sigma Aldrich), Anti-KLF7 Ab197690(Abcam), and Anti-PBX1 SAB2501446(Sigma Aldrich), was used at dilution of 1:200, 1:100, 1:100, and 1:100 respectively. After repeated washes, and slides were incubated with secondary antibody for 30 minutes at room temperature. Slides were rewashed in PBST buffer several times. After counterstaining with DAPI, slides were mounted with Antifade mounting solution (VectashieldTM) and then the images were taken for imaging in Zeiss AxioImager Z1 (EBL) and then processed using ImageJ software (version 1.53c).

Supplementary Table 1: Markers used for annotation of cell types.

	CellTypes	Markers	Reference
1	Activated T cells	CD69	24
2	Horizontal Cells (HC)	ONECUT1	25
3	Amacrine Cells (AC)	TFAP2A	26
4	Monocytes	CD14,CD74	27
5	Ciliary body cells	AQP1	28
6	Cone photoreceptors (Cone PR)	ARR3	29
7	Rod photoreceptors (Rod PR)	RHO	30
8	TGFBI high Corneal epithelial cells	ANXA1, TGFBI	31<br>32
9	ELF3 High Corneal Epithelial cells	ELF3, KRT12	33
10	Putative stem cells	PAX6, MSX1,MSX2	34 35
11	COL9A1-high ciliary body cells	PAX6, CPAMD8, COL9A1	36
12	Pigmented ciliary body cells.	DCT, PAX6, MLANA	37
13	Melanocyte	MLANA	38
14	Mueller glial cells (MG)	CRABP1	39
15	Ribosomal-genes high iris cells	RPL34, RPL17	40
16	Retinal ganglion cells (RGC)	SNCG	41
17	Retinal Pigmented Epithelium cells (RPE)	RPE65	38
18	Schwann cells	CD9, LGI4	42 43
19	Smooth Muscle Cells (SMCs)	MYH11, MYL9	44

20	Fibroblasts (FB)	APOD,DCN	45
21	Bipolar cells (BC)	GRM6, PCP2	46
22	OFF Biopolar cells (OFF BCs)	GRIK1	47
23	Rod Bipolar cells (Rod BP)	PRKCA	48
24	Microglia (MG)	APOE, B2M	49
25	Conjuctival cells	KRT4, KRT19	50
26	MEG3-high fibroblast cells	MEG3	51
27	WIF1- high fibroblast cells	WIF1	52
28	CRYAA- high ciliary body cells	CRYAA	53

Supplementary References

1 Zheng, G. X. *et al.* Massively parallel digital transcriptional profiling of single cells. *Nat Commun* **8**, 14049, doi:10.1038/ncomms14049 (2017).

2 Stuart, T. *et al.* Comprehensive Integration of Single-Cell Data. *Cell* **177**, 1888-1902.e1821, doi:10.1016/j.cell.2019.05.031 (2019).

3 Wolock, S. L., Lopez, R. & Klein, A. M. Scrublet: Computational Identification of Cell Doublets in Single-Cell Transcriptomic Data. *Cell Syst* **8**, 281-291.e289, doi:10.1016/j.cels.2018.11.005 (2019).

4 Lukowski, S. W. *et al.* A single-cell transcriptome atlas of the adult human retina. *Embo j* **38**, e100811, doi:10.15252/embj.2018100811 (2019).

5 Peng, Y. R. *et al.* Molecular Classification and Comparative Taxonomics of Foveal and Peripheral Cells in Primate Retina. *Cell* **176**, 1222-1237.e1222, doi:10.1016/j.cell.2019.01.004 (2019).

6 Macosko, E. Z. *et al.* Highly Parallel Genome-wide Expression Profiling of Individual Cells Using Nanoliter Droplets. *Cell* **161**, 1202-1214, doi:10.1016/j.cell.2015.05.002 (2015).

7 Hoang, T. *et al.* Gene regulatory networks controlling vertebrate retinal regeneration. *Science* **370**, doi:10.1126/science.abb8598 (2020).

8 Menon, M. *et al.* Single-cell transcriptomic atlas of the human retina identifies cell types associated with age-related macular degeneration. *Nat Commun* **10**, 4902, doi:10.1038/s41467-019-12780-8 (2019).

9 Satija, R., Farrell, J. A., Gennert, D., Schier, A. F. & Regev, A. Spatial reconstruction of single-cell gene expression data. *Nat Biotechnol* **33**, 495-502, doi:10.1038/nbt.3192 (2015).

10 Zhou, Y. *et al.* Metascape provides a biologist-oriented resource for the analysis of systems-level datasets. *Nat Commun* **10**, 1523, doi:10.1038/s41467-019-09234-6 (2019).

11 Weng, M.-P. & Liao, B.-Y. modPhEA: model organism Phenotype Enrichment Analysis of eukaryotic gene sets. *Bioinformatics* **33**, 3505-3507, doi:10.1093/bioinformatics/btx426 (2017).

12 Aran, D. *et al.* Reference-based analysis of lung single-cell sequencing reveals a transitional profibrotic macrophage. *Nature Immunology* **20**, 163-172, doi:10.1038/s41590-018-0276-y (2019).

13 Stein-O'Brien, G. L. *et al.* Decomposing cell identity for transfer learning across cellular measurements, platforms, tissues, and species. *Cell systems* **8**, 395-411. e398 (2019).

14 Vento-Tormo, R. *et al.* Single-cell reconstruction of the early maternal-fetal interface in humans. *Nature* **563**, 347-353, doi:10.1038/s41586-018-0698-6 (2018).

15 Teschendorff, A. E., Morabito, S. J., Kessenbrock, K. & Meyer, K. Integrated single-cell potency and expression landscape in mammary epithelium reveals novel bipotent-like cells associated with breast cancer risk. *bioRxiv*, 496471, doi:10.1101/496471 (2018).

16 La Manno, G. *et al.* RNA velocity of single cells. *Nature* **560**, 494-498, doi:10.1038/s41586-018-0414-6 (2018).

17 Grove, J. & Marsh, M. in *J Cell Biol* Vol. 195 1071-1082 (2011).

18 Bhattacharjee, S. Recent advances in host–virus interactomics during entry and infection. *Virus Adaptation and Treatment* **7**, 57-66 (2015).

19 van Dijk, D. *et al.* Recovering Gene Interactions from Single-Cell Data Using Data Diffusion. *Cell* **174**, 716-729.e727, doi:10.1016/j.cell.2018.05.061 (2018).

20 Aibar, S. *et al.* SCENIC: single-cell regulatory network inference and clustering. *Nat Methods* **14**, 1083-1086, doi:10.1038/nmeth.4463 (2017).

21 Imrichová, H., Hulselmans, G., Kalender Atak, Z., Potier, D. & Aerts, S. i-cisTarget 2015 update: generalized cis-regulatory enrichment analysis in human, mouse and fly. *Nucleic Acids Res* **43**, W57-64, doi:10.1093/nar/gkv395 (2015).

22 Shannon, P. *et al.* in *Genome Res* Vol. 13 2498-2504 (2003).

23 Lee, J. *et al.* Defined Conditions for Differentiation of Functional Retinal Ganglion Cells From Human Pluripotent Stem Cells. *Invest Ophthalmol Vis Sci* **59**, 3531-3542, doi:10.1167/iovs.17-23439 (2018).

24 Ziegler, S. F., Ramsdell, F. & Alderson, M. R. The activation antigen CD69. *Stem Cells* **12**, 456-465, doi:10.1002/stem.5530120502 (1994).

25 Klimova, L., Antosova, B., Kuzelova, A., Strnad, H. & Kozmik, Z. Onecut1 and Onecut2 transcription factors operate downstream of Pax6 to regulate horizontal cell development. *Dev Biol* **402**, 48-60, doi:10.1016/j.ydbio.2015.02.023 (2015).

26 Jin, K. *et al.* in *Molecular brain* Vol. 8 (2015).

27 Sampath, P., Moideen, K., Ranganathan, U. D. & Bethunaickan, R. Monocyte Subsets: Phenotypes and Function in Tuberculosis Infection. *Front Immunol* **9**, 1726, doi:10.3389/fimmu.2018.01726 (2018).

28 Yamaguchi, Y., Watanabe, T., Hirakata, A. & Hida, T. Localization and ontogeny of aquaporin-1 and -4 expression in iris and ciliary epithelial cells in rats. *Cell Tissue Res* **325**, 101-109, doi:10.1007/s00441-005-0122-z (2006).

29 Khalili, S. *et al.* Induction of rod versus cone photoreceptor-specific progenitors from retinal precursor cells. *Stem Cell Res* **33**, 215-227, doi:10.1016/j.scr.2018.11.005 (2018).

30 Blackshaw, S., Fraioli, R. E., Furukawa, T. & Cepko, C. L. Comprehensive analysis of photoreceptor gene expression and the identification of candidate retinal disease genes. *Cell* **107**, 579-589, doi:10.1016/s0092-8674(01)00574-8 (2001).

31 Mimura, K. K., Tedesco, R. C., Calabrese, K. S., Gil, C. D. & Oliani, S. M. in *Mol Vis* Vol. 18 1583-1593 (2012).

32 Han, Y. P., Sim, A. J., Vora, S. C. & Huang, A. J. W. in *Invest Ophthalmol Vis Sci* Vol. 52 8401-8406 (2011).

33 Luk, I. Y., Reehorst, C. M. & Mariadason, J. M. in *Molecules* Vol. 23 (2018).

34 Osumi, N., Shinohara, H., Numayama-Tsuruta, K. & Maekawa, M. Concise review: Pax6 transcription factor contributes to both embryonic and adult neurogenesis as a multifunctional regulator. *Stem Cells* **26**, 1663-1672, doi:10.1634/stemcells.2007-0884 (2008).

35 Belanger, M. C., Robert, B. & Cayouette, M. Msx1-Positive Progenitors in the Retinal Ciliary Margin Give Rise to Both Neural and Non-neural Progenies in Mammals. *Dev Cell* **40**, 137-150, doi:10.1016/j.devcel.2016.11.020 (2017).

36 Cheong, S. S. *et al.* in *Am J Hum Genet* Vol. 99 1338-1352 (2016).

37 Jiao, Z. *et al.* Dopachrome tautomerase (Dct) regulates neural progenitor cell proliferation. *Dev Biol* **296**, 396-408, doi:10.1016/j.ydbio.2006.06.006 (2006).

38 Voigt, A. P. *et al.* Single-cell transcriptomics of the human retinal pigment epithelium and choroid in health and macular degeneration. *Proc Natl Acad Sci U S A* **116**, 24100-24107, doi:10.1073/pnas.1914143116 (2019).

39 Todd, L., Suarez, L., Quinn, C. & Fischer, A. J. Retinoic Acid-Signaling Regulates the Proliferative and Neurogenic Capacity of Muller Glia-Derived Progenitor Cells in the Avian Retina. *Stem Cells* **36**, 392-405, doi:10.1002/stem.2742 (2018).

40 Prakash, V. *et al.* in *Nat Commun* Vol. 10 (2019).

41 Soto, I. *et al.* Retinal ganglion cells downregulate gene expression and lose their axons within the optic nerve head in a mouse glaucoma model. *J Neurosci* **28**, 548-561, doi:10.1523/jneurosci.3714-07.2008 (2008).

42 Banerjee, S. A. & Patterson, P. H. Schwann cell CD9 expression is regulated by axons. *Mol Cell Neurosci* **6**, 462-473, doi:10.1006/mcne.1995.1034 (1995).

43 Xue, S. *et al.* Loss-of-Function Mutations in LGI4, a Secreted Ligand Involved in Schwann Cell Myelination, Are Responsible for Arthrogryposis Multiplex Congenita. *Am J Hum Genet* **100**, 659-665, doi:10.1016/j.ajhg.2017.02.006 (2017).

44 Chakraborty, R. *et al.* Promoters to Study Vascular Smooth Muscle. *Arterioscler Thromb Vasc Biol* **39**, 603-612, doi:10.1161/atvbaha.119.312449 (2019).

45 Cui, Y. *et al.* Single-Cell Transcriptome Analysis Maps the Developmental Track of the Human Heart. *Cell Rep* **26**, 1934-1950.e1935, doi:10.1016/j.celrep.2019.01.079 (2019).

46 Morgan, J. L., Dhingra, A., Vardi, N. & Wong, R. O. Axons and dendrites originate from neuroepithelial-like processes of retinal bipolar cells. *Nat Neurosci* **9**, 85-92, doi:10.1038/nn1615 (2006).

47 Murphy, D. P., Hughes, A. E., Lawrence, K. A., Myers, C. A. & Corbo, J. C. in *eLife* Vol. 8 (2019).

48 Ruether, K. *et al.* in *Invest Ophthalmol Vis Sci* Vol. 51 6051-6058 (2010).

49 Kang, S. S. *et al.* Microglial translational profiling reveals a convergent APOE pathway from aging, amyloid, and tau. *J Exp Med* **215**, 2235-2245, doi:10.1084/jem.20180653 (2018).

50 Harun, M. H. *et al.* Human forniceal region is the stem cell-rich zone of the conjunctival epithelium. *Hum Cell* **26**, 35-40, doi:10.1007/s13577-011-0025-0 (2013).

51 Wang, Y., Wang, J., Wei, L. J., Zhu, D. M. & Zhang, J. S. Biological function and mechanism of lncRNA-MEG3 in Tenon's capsule fibroblasts proliferation: By MEG3-Nrf2 protein interaction. *Biomed Pharmacother* **87**, 548-554, doi:10.1016/j.biopha.2016.12.040 (2017).

52 Park, T. J. *et al.* Wnt inhibitory factor (WIF)-1 promotes melanogenesis in normal human melanocytes. *Pigment Cell Melanoma Res* **27**, 72-81, doi:10.1111/pcmr.12168 (2014).

53 Wolf, L., Yang, Y., Wawrousek, E. & Cvekl, A. Transcriptional regulation of mouse alpha A-crystallin gene in a 148kb Cryaa BAC and its derivates. *BMC Dev Biol* **8**, 88, doi:10.1186/1471-213x-8-88 (2008).
