# Supplementary material for: Multi-species single-cell transcriptomic analysis of ocular compartment regulons
Source: Nat Commun. 2021 Sep 28;12:5675. doi: 10.1038/s41467-021-25968-8 (PMC8478974; doi:10.1038/s41467-021-25968-8)
Supplement: Supplementary file 3 — Description of Supplementary files [file 41467_2021_25968_MOESM3_ESM.docx]

**Description of Additional Supplementary Files**

File Name: Supplementary Data 1.

Description: One-sided Wilcoxon rank-sum test p-values for the markers of cell types in retina across donors.

File Name: Supplementary Data 2.

Description: One-sided Wilcoxon rank-sum test p-values for the markers of cell types in choroid/sclera, iris/ciliary body and cornea.

File Name: Supplementary Data 3.

Description: Correlation of cell-type specific marker-gene expression with individual patterns.

File Name: Supplementary Data 4.

Description: p-value for the likelihood of cell-type specificity of a given receptor-ligand complex. It is calculated based on the proportion of the means that are as high as or higher than the actual mean.

File Name: Supplementary Data 5.

Description: List of transcription factors enriched in a cell type derived from pairwise correlation scores of SCENIC regulon activity and MAGIC imputation values.

File Name: Supplementary Data 6.

Description: -log(p values) of GO terms specific to a cell type. P-value is the probability or chance of seeing at least x number of genes out of the total n genes in the list annotated to a particular GO term, given the proportion of genes in the whole genome that are annotated to that GO Term.
