# Supplementary material for: Multi-species single-cell transcriptomic analysis of ocular compartment regulons
Source: Nat Commun. 2021 Sep 28;12:5675. doi: 10.1038/s41467-021-25968-8 (PMC8478974; doi:10.1038/s41467-021-25968-8)
Supplement: Supplementary file 11 — Reporting Summary [file 41467_2021_25968_MOESM11_ESM.pdf]

nature research

Corresponding author(s): Yuin Han Loh

Last updated by author(s): 22/7/2021

Reporting Summary

Nature Research wishes to improve the reproducibility of the work that we publish. This form provides structure for consistency and transparency in reporting. For further information on Nature Research policies, see our Editorial Policies and the Editorial Policy Checklist.

Statistics

For all statistical analyses, confirm that the following items are present in the figure legend, table legend, main text, or Methods section.

n/a	Confirmed	
☐	☒	The exact sample size (n) for each experimental group/condition, given as a discrete number and unit of measurement
☐	☒	A statement on whether measurements were taken from distinct samples or whether the same sample was measured repeatedly
☐	☒	The statistical test(s) used AND whether they are one- or two-sided<br>*Only common tests should be described solely by name; describe more complex techniques in the Methods section.*
☐	☒	A description of all covariates tested
☐	☒	A description of any assumptions or corrections, such as tests of normality and adjustment for multiple comparisons
☒	☐	A full description of the statistical parameters including central tendency (e.g. means) or other basic estimates (e.g. regression coefficient) AND variation (e.g. standard deviation) or associated estimates of uncertainty (e.g. confidence intervals)
☐	☒	For null hypothesis testing, the test statistic (e.g. F, t, r) with confidence intervals, effect sizes, degrees of freedom and P value noted<br>*Give P values as exact values whenever suitable.*
☒	☐	For Bayesian analysis, information on the choice of priors and Markov chain Monte Carlo settings
☒	☐	For hierarchical and complex designs, identification of the appropriate level for tests and full reporting of outcomes
☐	☒	Estimates of effect sizes (e.g. Cohen's d, Pearson's r), indicating how they were calculated

Our web collection on statistics for biologists contains articles on many of the points above.

Software and code

Policy information about availability of computer code

Data collection	Single cell RNA sequencing data was generated using Illumina Hiseq 4000 according to the manufacture's instructions.
Data analysis	For the single cell data, we used the publicly available Cell Ranger version >2.1.1. pipeline (10X Genomics), Seurat R package >v3.0.1, Monocle R package v2.12.0, Rmagic R package v2.0.3, nichenetr R package v0.1.0, SCENIC R package v1.1.2.2, LandSCENT R package v0.99.3, SingleR R package v1.4.1, CoGAPS R package v3.6.0, ProjectR R package v1.5.0, Scrublet python package v0.2.1, CellPhoneDB python package v2.1.5, velocyto python package v0.17.17, Cytoscape (version 3.7.1), modPhEA (http://evol.nhri.org.tw/phenome2/) and metascape (https://metascape.org/) for analysis. All the codes used in this study are available. The schematic workflow was illustrated using BioRender (https://biorender.com). ImageJ (version 1.53c) was used to create RNA FISH images by using stacking to create a single image.

For manuscripts utilizing custom algorithms or software that are central to the research but not yet described in published literature, software must be made available to editors and reviewers. We strongly encourage code deposition in a community repository (e.g. GitHub). See the Nature Research guidelines for submitting code & software for further information.

Data

Policy information about availability of data

All manuscripts must include a data availability statement. This statement should provide the following information, where applicable:

- Accession codes, unique identifiers, or web links for publicly available datasets
- A list of figures that have associated raw data
- A description of any restrictions on data availability

Single-cell RNA-seq data (human, pig and zebrafish eyes) have been deposited in the Gene Expression Omnibus (GEO) under the accession code GSE147979. Previously published single-cell RNA-seq data that were reanalysed here are available in the GEO or ArrayExpress under the accession codes GSE118480 (macaque retina cells), GSE63472 (mouse retina cells), GSE135406 (zebrafish retina cells), GSE137537 (human retina cells) and E-MTAB-7316 (human macula/periphery cells).

scRNA-seq data can be queried interactively at the Single Cell Portal (SCP) under the accession code SCP1311 (primary tissues) and, SCP1386, SCP1387, SCP1388, SCP1389, SCP1390, SCP1391 (in vitro RGC differentiation).

Field-specific reporting

Please select the one below that is the best fit for your research. If you are not sure, read the appropriate sections before making your selection.

- [x] Life sciences
- [ ] Behavioural & social sciences
- [ ] Ecological, evolutionary & environmental sciences

For a reference copy of the document with all sections, see nature.com/documents/nr-reporting-summary-flat.pdf

Life sciences study design

All studies must disclose on these points even when the disclosure is negative.

Sample size	No predetermined sample sizes were performed. The samples having no known ocular pathology were chosen in random. We analyzed different tissues from ocular compartment from six individuals for 10x genomics single cell RNA seq platforms. The number of samples was sufficient to confidently annotate individual cell groups, and perform data analysis.
Data exclusions	In the methods, tissues from ocular compartments collected for the study were checked for indications of disease pathology. Low quality cells were filtered out during post-processing of samples.
Replication	At least two technical replicates for each target were used for RNA FISH studies (Fig 2c, d, e). Biological replicates couldn't be included due to unavailability of human retinal slides from different donors. However, to address this issue, we carried out Immunofluorescence studies for some of the targets whose antibodies were available (KLF7, PBX1 and SREBP2 in Fig 7b and SFig 7a) in non-human primate samples. We used different technique to validate similar results obtained by RNA FISH and scRNAseq studies. RNA FISH experiments were done as an additional confirmation for scRNAseq datasets and lack of biological replicates in RNA FISH experiments (for Fig 2c, d, e) doesn't affect the studies. All replication attempts were successful.
Randomization	Tissues were not randomized. However, co-variates were controlled by integration methods in Seurat v3 software. It clustered single-cell RNA sequencing data based on the common cell states across replicates.
Blinding	Investigators were blinded to group allocation during sample collection and analysis: the sample collection and analysis were done by separate researchers.

Reporting for specific materials, systems and methods

We require information from authors about some types of materials, experimental systems and methods used in many studies. Here, indicate whether each material, system or method listed is relevant to your study. If you are not sure if a list item applies to your research, read the appropriate section before selecting a response.

Materials & experimental systems

n/a	Involved in the study	
☐	☒	Antibodies
☐	☒	Eukaryotic cell lines
☒	☐	Palaeontology and archaeology
☐	☒	Animals and other organisms
☐	☒	Human research participants
☒	☐	Clinical data
☒	☐	Dual use research of concern

Methods

n/a	Involved in the study	
☒	☐	ChIP-seq
☒	☐	Flow cytometry
☒	☐	MRI-based neuroimaging

Antibodies

Antibodies used	Anti-PKCA(H-7) Sc-8393(Santa Cruz Biotechnology), Anti-TUJ1 Ab18207(Abcam), Anti-SREBP2 AV09037(Sigma Aldrich), Anti-KLF7 Ab197690(Abcam), Anti-PBX1 SAB2501446(Sigma Aldrich), Anti-Vimentin Ab24525(Abcam)
Validation	The Anti-PKCA(H-7) Sc-8393(Santa Cruz Biotechnology)<br>https://www.scbt.com/p/pkc-alpha-antibody-h-7<br>Validated for IF in K562 cells by the manufacturer. This antibody has been cited in 270 publications. It was used at dilution of 1:100.<br><br>Anti-TUJ1 Ab18207(Abcam)<br>https://www.abcam.com/beta-iii-tubulin-antibody-neuronal-marker-ab18207.html<br>Validated for IF in SK-N-SH (Human neuroblastoma cell line) cells by the manufacturer. This antibody has been cited in 332 publications. It was used at dilution of 1:200.

Anti-SREBP2 AV09037(Sigma Aldrich)
https://www.sigmaaldrich.com/SG/en/product/sigma/av09037
Validated for WB by the manufacturer. They are also validated in our lab in H9 embryonic stem cells. It was used at dilution of 1:100.

Anti-KLF7 Ab197690(Abcam)
https://www.abcam.com/klf7-antibody-ab197690.html
Validated for IHC in paraffin-embedded Human thyroid cancer tissue by manufacturer. Validated by https://www.ncbi.nlm.nih.gov/pmc/articles/PMC5704477/ for IF in mouse retina. It was used at dilution of 1:100.

Anti-PBX1 SAB2501446(Sigma Aldrich)
https://www.sigmaaldrich.com/SG/en/product/sigma/sab2501446
Validated for WB and indirect ELISA by manufacturer. Validated in our lab using RNA FISH probes showing localization in overlapping regions of non-human primate retina. It was used at dilution of 1:100.

Anti-Vimentin Ab24525(Abcam)
https://www.abcam.com/vimentin-antibody-ab24525.html
Validated for IF in rat cerebral cortex by manufacturer. Validated by https://www.ncbi.nlm.nih.gov/pmc/articles/PMC7141751/ for IF in human retina. It was used at dilution of 1:500.

Eukaryotic cell lines

Policy information about cell lines

Cell line source(s)	H9 [WA09, P35–50], WiCell, Madison, WI, USA
Authentication	The cells were obtained from WiCell Research Institute (WA). The authentication of cell lines performed by Wicell could be found at https://hpscreg.eu/docs/uploads//certificate_of_analysis/0f9cf0fcf67f00bdaf9bc777495146d9.pdf
Mycoplasma contamination	The cultures were tested negative for mycoplasma contamination by PCR.
Commonly misidentified lines (See ICLAC register)	None

Animals and other organisms

Policy information about studies involving animals; ARRIVE guidelines recommended for reporting animal research

Laboratory animals	Male Zebrafish AB wildtype strain, 3 months old.
Wild animals	The study did not involve wild animals.
Field-collected samples	Pig eye samples were collected from Agri-Food and Veterinary Authority of Singapore approved abattoirs. The conditions of the abattoir was followed according to WHOLE MEAT AND FISH ACT enforced by Singapore Food Agency.
Ethics oversight	All experiments in zebrafish was approved by Singapore National Advisory on Laboratory Animal Research.

Note that full information on the approval of the study protocol must also be provided in the manuscript.

Human research participants

Policy information about studies involving human research participants

Population characteristics	We selected six individuals for single- cell RNA sequencing and a single donor for RNA fluoroscence in situ hybridization studies. All of such seven individuals were confirmed to have no known ocular pathology.
Recruitment	No donors were recruited. Informed consent was obtained, and the postmortem eyes were obtained from autopsies from donors aging between 28 and 84 years old from the Eye Bank for Sight Restoration, New York, NY, USA.
Ethics oversight	Studies were conducted under IRB oversight. All tissue was de-identified prior to delivery to the eye bank, which is conducted under an approved IRB protocol following US HIPAA privacy law. Tissue was then sent to the Blenkinsop lab for dissection. All de-identified medical information given was under the consent of donors. Single cell RNAseq transcripts were predicted by poly -A tag sequencing of the first 50 bases and therefore no personal genetic information was collected that may be

identifiable or traced back to donors.

Note that full information on the approval of the study protocol must also be provided in the manuscript.
